# Plastoglobules: A metabolic hub for nitrogen assimilation

**DOI:** 10.1016/j.xplc.2026.101974

**Published:** 2026-06-17

**Authors:** Wei Wang, Chengcai Chu

**Affiliations:** 1 Guangdong Basic Research Center of Excellence for Precise Breeding of Future Crops and Guangdong Laboratory for Lingnan Modern Agriculture, College of Agriculture, South China Agricultural University, Guangzhou 510642, China; 2 Key Laboratory for Enhancing Resource Use Efficiency of Crops in South China, Ministry of Agriculture and Rural Affairs, South China Agricultural University, Guangzhou 510642, China; 3 Guangdong Provincial Key Laboratory of Plant Molecular Breeding, College of Agriculture, South China Agricultural University, Guangzhou 510642, China

## Main text

Nitrogen (N) is an essential macronutrient for crop growth and productivity. Improving nitrogen–use efficiency (NUE) is critical for global food security and sustainable agriculture. NUE is a complex trait shaped by N acquisition, assimilation, and remobilization (Wang et al., 2026). Among these, N assimilation converts inorganic N into organic forms, such as amino acids, that are essential for cellular metabolism (Liu et al., 2022). In brief, nitrate is reduced to nitrite in the cytoplasm by nitrate reductase, nitrite is then imported into plastids and reduced to ammonium by nitrite reductase (NiR). The resulting ammonium, together with ammonium taken up from the soil by roots, is rapidly incorporated into organic N through the glutamine synthetase/glutamate synthase cycle (Liu et al., 2022). Despite the well-established role of plastids in N assimilation, the exact subcompartment in which this process occurs has remained unclear.

Land plants exhibit diverse plastid morphologies, but all plastids share common features: a double-membrane envelope, an aqueous stroma containing proteins and ribosomes, nucleoids, plastoglobules (PGs), vesicles, and an internal membrane system (Solymosi et al., 2018). PGs are lipid droplet-like structures anchored to thylakoid membranes and enclosed by a lipid monolayer (Austin et al., 2006). They participate in membrane repair, remodeling, and resynthesis; protect against oxidative stress; and store carotenoids, tocopherols, and lipids (Solymosi et al., 2018). PG abundance and size vary with plastid type, developmental stage, and environmental conditions (e.g., nutrient deficiency). However, the precise role of PGs in nutrient utilization remains unclear. In photosynthetic tissues, chloroplasts are the primary site of both PG localization and N assimilation.

In a recent breakthrough, Chen et al. (2026) demonstrated that PGs function as a central metabolic hub for N assimilation in mesophyll chloroplasts in maize, a C_4_ species. Using transmission electron microscopy together with transient expression of the PG marker ZmPSY3-mCherry, they found that PG number and size both increased with N availability in mesophyll chloroplasts, independently of chloroplast size. This response was absent in bundle sheath chloroplasts. Similar N-induced increases in PG abundance were observed in four C_3_ species (soybean, tobacco, rice, and wheat), indicating that PG dynamics in mesophyll chloroplasts reflect a conserved structural response in both C_3_ and C_4_ plants.

To investigate the biochemical basis of PG-mediated N responsiveness, the authors isolated highly purified PGs from maize mesophyll chloroplasts and performed liquid chromatography–tandem mass spectrometry. Proteomic profiling identified three major categories of PG-associated proteins: structural proteins, enzymes involved in secondary metabolism, and proteins associated with carbon (C) and N metabolism. The first two categories largely corresponded to known PG components. Subcellular localization analyses of 13 representative proteins across all three categories confirmed their co-localization with PGs, validating the proteomics data. Remarkably, nitrite reductase 2 (ZmNIR2) and glutamine synthetase 1 (ZmGLN1) were identified as key PG constituents, directly linking PGs to N assimilation.

The maize genome contains two *ZmNIR* and six *ZmGLN* genes, but only ZmNIR2 and ZmGLN1 were specifically targeted to PGs. Truncation experiments mapped the chloroplast transit peptide and the hydrophobic regions required for PG association, thereby revealing their targeting mechanisms. Both genes were predominantly expressed in leaves. Genetic evidence confirmed their functional importance, as *zmnir2* and *zmgln1* mutants exhibited reduced biomass and impaired responses to N.

ZmGLN1 forms a stable decamer in plants, consistent with the oligomeric states of maize cytosolic ZmGLN5 and rice chloroplastic OsGS2 (Zhang et al., 2024). Because ZmNIR2 and ZmGLN1 catalyze sequential steps in the conversion of nitrite to glutamine, the authors tested whether these two proteins interact with each other. Multiple assays confirmed a weak interaction, indicating that these enzymes assemble into a metabolon within PGs that spatially couples nitrite reduction with ammonium assimilation to maximize metabolic efficiency. Notably, increasing N levels promoted the localization of both enzymes to PGs, raising the question of whether N directly enhances their interaction.

An analysis of natural variation revealed that *ZmNIR2 undergoes* alternative splicing to produce two transcripts, *ZmNIR2*^*T1*^ and *ZmNIR2*^*T2*^. ZmNIR2^T2^ lacks the N-terminal chloroplast transit peptide present in ZmNIR2^T1^. As expected, ZmNIR2^T1^ localized to PGs, whereas ZmNIR2^T2^ failed to enter chloroplasts. Inbred maize lines with higher proportions of the *ZmNIR2*^*T1*^ transcript exhibited greater N-dependent increases in biomass and glutamine content, identifying ZmNIR2^T1^ as the favorable isoform. Notably, teosinte accessions uniformly expressed high levels of *ZmNIR2*^*T1*^, whereas some modern maize lines exhibited high *ZmNIR2*^*T2*^ expression. Correlation analyses across diverse maize populations may help dissect potential trait trade-offs resulting from linkage drag or pleiotropy during domestication. Field experiments showed that *ZmNIR2*^*T1*^ overexpression increased plant height and biomass, underscoring its potential for improving NUE.

**Figure 1:**
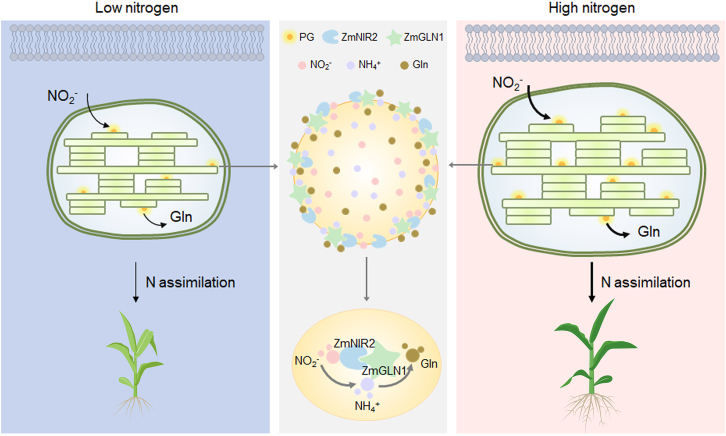
Model depicting maize chloroplast PGs as a hub for primary nitrogen assimilation that underlies nitrogen-promoted growth. ZmNIR2 and ZmGLN1 assemble into a PG-localized metabolon that couples nitrite reduction with ammonium assimilation. Compared with low-nitrogen conditions, high nitrogen increases PG number and size, thereby enhancing nitrogen assimilation and promoting vegetative growth. The width of the black arrows represents the relative strength of the regulatory or metabolic flux.

In summary, Chen et al. (2026) establish PGs as a central hub for primary N assimilation (Figure 1). The PG-localized enzymes ZmNIR2 and ZmGLN1 interact to form a metabolon, thereby maximizing assimilation efficiency. In addition, natural splicing variation in *ZmNIR2* in cultivated germplasm generates a PG-targeted isoform (ZmNIR2^T1^) that enhances NUE.

This work opens several exciting avenues for future research. First, how does N availability regulate PG proliferation? Abiotic stresses (e.g., drought, heat, and salinity) increase oxidative stress, which in turn can trigger thylakoid breakdown and PG expansion (Besagni and Kessler, 2013; Nam et al., 2022; Coulon et al., 2024). In contrast, high N levels typically reduce reactive oxygen species (ROS) levels (Yuan et al., 2026), suggesting that N-induced PG expansion is not mediated by oxidative stress. Interestingly, *ZmNIR2*^*T1*^ overexpression increased both PG number and size, hinting at a feedback loop in which the enzyme or its products promote PG biogenesis. N may also drive PG proliferation by directly upregulating structural scaffold proteins, such as fibrillins, and activating lipid biosynthetic genes. Determining how N drives PG biogenesis could reveal whether PG formation itself serves as a higher-order regulatory layer for tuning NUE.

Second, although PGs serve as dedicated platforms for the ZmNIR2–ZmGLN1 metabolon, the biological significance of this dual targeting remains unclear. Experimentally uncoupling PG localization from enzyme interaction (e.g., by mutating hydrophobic anchoring regions without disrupting physical associations) could test whether PG targeting *per se* is required for optimal N assimilation. Such approaches may reveal whether PGs provide a unique microenvironment that brings substrates (nitrite and ammonium) and enzymes (ZmNIR2 and ZmGLN1) together for efficient metabolon function. Other PG-resident metabolites and local physicochemical conditions may also contribute, at least in part, to metabolon activity. Furthermore, because PG-mediated metabolic compartmentalization may represent an evolutionarily robust strategy for optimizing N use in land plants, it would be intriguing to examine whether PG targeting of NiR and glutamine synthetase has evolved differentially across species and whether this correlates with interspecific variation in N demand.

Finally, and most importantly, Chen et al. (2026) fundamentally revise the conventional view of PGs by demonstrating that these structures function as a central metabolic compartment for N assimilation, one of the most important metabolic processes in plants (Figure 1). Their work provides a blueprint for dissecting plant metabolism at suborganellar resolution. By identifying where key enzymes operate within organelles, they advance our understanding of this process from the organellar level to the suborganellar level. This study illustrates that the subcellular organization of metabolism is just as critical as the catalytic efficiency of individual enzymes. Given the numerous biochemical reactions that occur within chloroplasts, mitochondria, and the endoplasmic reticulum, the framework established here opens new avenues for precise suborganellar engineering to improve crop performance.

## Funding

This work was supported by the National Natural Science Foundation of China (32302655 and 32130095).

## Acknowledgments

No conflict of interest is declared.
